# Hypothalamic–pituitary–adrenal axis suppression by inhaled or nasal corticosteroids in HIV-infected patients

**DOI:** 10.1007/s11096-020-00995-5

**Published:** 2020-03-05

**Authors:** Femke Besemer, Cornelis Kramers, Kees Brinkman, Ad R. M. M. Hermus, Antonius E. van Herwaarden, David M. Burger

**Affiliations:** 1 Department of Internal Medicine, Radboudumc Nijmegen, Nijmegen, The Netherlands; 2 Department of Internal Medicine, Onze Lieve Vrouwe Gasthuis (OLVG), Amsterdam, The Netherlands; 3 Department of Laboratory Medicine, Radboudumc Nijmegen, Nijmegen, The Netherlands; 4 Pharmacy Radboudumc Nijmegen, Nijmegen, The Netherlands; 5 Present Address: Nij Smellinge Ziekenhuis, Drachten, The Netherlands; 6 Internal Medicine, Nij Smellinghe, Compagnonsplein 1, 9202 NN Drachten, the Netherlands

**Keywords:** Adrenal insufficiency, Antiretroviral therapy, Drug interactions, Inhaled corticosteroid, Nasal corticosteroid

## Abstract

*Background* Inhaled or nasal corticosteroids can cause suppression of the hypothalamic–pituitary–adrenal (HPA) axis. Early detection is important because this suppression can be associated with significant morbidity. *Objective *To explore the adverse effect of hypothalamic–pituitary–adrenal suppression by local corticosteroids in HIV-infected patients. *Method *Ambulatory HIV-infected patients were selected if they used both antiretroviral treatment and inhaled or nasal corticosteroid. Suppression of hypothalamic–pituitary–adrenal axis was defined as a morning plasma cortisol below 80 nmol/L or a cortisol below 550 nmol/L during a 250 mcg adrenocorticotropic hormone-stimulation test. *Results *Twelve patients were tested; four of them were taking a CYP3A4 inhibitor. All patients had a normal morning plasma cortisol. Suppression of the hypothalamic–pituitary–adrenal axis during the ACTH stimulation test was identified in three of the twelve patients. None of these three individuals were taking a CYP3A4 inhibitor. *Conclusion *Hypothalamic–pituitary–adrenal axis suppression is frequently identified in patients on inhaled or nasal corticosteroids. CYP3A4 inhibitors such as ritonavir or cobicistat may increase the chance of this adverse effect. In this study we did not identify HPA axis suppression in patients taking CYP3A4 inhibitors. This may be related to the fact that 2 of these 4 patients used beclomethasone, a corticosteroid not metabolized by CYP3A4.

*ClinicalTrials.gov Identifier* NCT02501486.

## Impacts on practice

- Drugs that are expected to act locally can result in systemic adverse effects.
- Some adverse effects are difficult to detect but can result in poor outcomes. Hypothalamic–pituitary–adrenal axis suppression is an example of this kind of adverse effect.

## Introduction

It is known that systemic corticosteroids can give symptoms of hypercortisolism and also lead to suppression of the hypothalamic–pituitary–adrenal (HPA)-axis. The prevalence of HPA axis suppression caused by local corticosteroids is unclear. A meta-analysis found HPA axis suppression in 4.2% of patients using nasal and 7.8% of patients using inhaled corticosteroids [1] Recognition of HPA axis suppression in this context is difficult: a patient can have clinical symptoms of hypercortisolism, while endogenous cortisol production is low. Stopping treatment with a local corticosteroid can lead to an adrenal crisis. An adrenal crisis can result in symptoms including nausea, vomiting, abdominal pain and even coma.

The occurrence of adrenal insufficiency and iatrogenic hypercortisolism have been reported several times in individuals taking a combination of inhaled or nasal fluticasone and ritonavir (an HIV protease inhibitor and strong CYP 3A4 inhibitor) [2]. Fluticasone has pharmacokinetic features which might explain the high chance for interaction: It is a CYP3A substrate and has a long glucocorticoid-receptor binding half-life [3]. Therefore the combination of fluticasone and ritonavir or cobicistat is discouraged [4]. Instead, it is advised that beclomethasone, a corticosteroid which is not a CYP3A4 substrate is used.

## Aim of the study

The main aim of this study was to examine how often asymptomatic HIV-infected patients have HPA axis suppression if they use nasal or inhaled corticosteroids. The secondary aim was to explore whether HPA axis suppression was seen more often in patients taking a booster (ritonavir or cobicistat, which are CYP3A4 inhibitors).

## Ethics approval

The study was conducted according to the principles of the Declaration of Helsinki and approved by the Medical Ethical Board of Radboud University Medical Centre Nijmegen (the Netherlands), ethics approval number; NL nr 51711.091.14. Informed consent was obtained from all participants. If a patient had HPA axis suppression the local corticosteroid was changed (if possible) and/or oral hydrocortisone was given.

## Methods

We selected adult HIV-infected patients who had used antiretroviral therapy and an inhaled or nasal corticosteroid for at least two weeks. These patients were treated for their HIV infection at outpatient departments of two different hospitals: Radboud University Medical Center Nijmegen (RUMC) and Onze Lieve Vrouwe Gasthuis (OLVG) in Amsterdam, both in the Netherlands. The patients in RUMC were identified by screening the electronic pharmacy database. The patients in OLVG were selected using the ATHENA database (Stichting HIV Monitoring Database).

Patients were excluded if one of the following conditions was present: known adrenal insufficiency, allergy to tetracosactide, Cushing’s syndrome, refractory heart failure, peptic ulcer, acute psychosis or if they had ever had an adrenocorticotropic hormone-stimulation test (ACTH-stimulation test) before. Furthermore patients were excluded if they had used topical corticosteroids or oral corticosteroids in the last three months or had received intramuscular or intra-articular corticosteroid injections in the last year. Women were excluded if they were pregnant, breast feeding or using oral contraception.

A short medical history and physical examination was performed. In order to detect signs of adrenal insufficiency and orthostatic blood pressure measurements were performed. Patients were screened for symptoms of hypercortisolism like moon face, ecchymosis or buffalo hump.

Plasma cortisol was measured in the morning between 8:00 and 9:30. On the same day an ACTH stimulation test was carried out. Patients were fasting overnight. A venous cannula was inserted and 250 microgram synthetic ACTH diluted in 100 mL NaCl 0.9% was infused in 10 min or the ACTH was injected intramuscularly. Blood samples were taken before and 60 and 90 min after the start of the ACTH infusion. The ACTH stimulation test was performed by research nurses.

Plasma cortisol was measured using an Electrochemiluminescence immunoassay (2nd gen) on a Modular E170 random access analyzer (Roche).

Suppression of HPA axis was defined as a morning plasma cortisol below 80 nmol/L or a cortisol below 550 nmol/L during the ACTH stimulation test [5].

## Results

In the RUMC the medication list of 405 patients was manually screened and of them nine had used inhaled corticosteroids and 16 of them had used nasal corticosteroids for at least 2 weeks in the past year. In the OLVG from the database 37 patients were selected with a recorded use of an inhalation steroid, however 14 of them already had stopped it. Twelve HIV-infected patients who were still using corticosteroids at the time of evaluation, gave consent to be included in the study (see also Table 1): eleven men and one woman, their mean age was 52 years (range 34–61 years). The mean duration of HIV-infection was 12.5 years (range 1–30 years). None of them had features of hypercortisolism, e.g. buffalo hump, ecchymosis or moon face. None of the individuals had orthostatic hypotension. Four individuals were using a booster [ritonavir (n = 2) or cobicistat (n = 2)]. The indication for the local corticosteroids was Chronic obstructive pulmonary disease (COPD) in four patients, asthma in one patient, both asthma and COPD in two patients. In the remaining five patients it was unclassified dyspnea (two individuals), unclassified allergy, oral cavity inflammation and hay fever) Six patients used inhaled corticosteroids, three nasal corticosteroids and three used both. The dosages of corticosteroids differed between 50 microg of nasal fluticasone and 800 microg of inhaled budesonide daily.

**Table 1:** Characteristics of the included patients

Characteristics of 12 tested patients	
*Demographics*	
Mean Age (range, SD) (years)	52 (34–61, SD 7.9)
Male sex	91.7%
Use of a booster	33.3%
Mean duration of HIV-infection (range, SD) (years)	12.5 (1–30, SD 8.52)
*Corticosteroid information*	
Kind of inhalation corticosteroid	5 beclomethasone, 1 budesonide, 1 ciclesonide, 2 fluticasone, 1 budesonide, 1 ciclesonide, 2 fluticasone
Mean duration of use (range, SD) (months)	65 (1–240, SD 69)
Kind of nasal corticosteroid	2 beclomethasone, 2 fluticasone, 2 mometasone, 2 fluticason, 2 mometason
Mean duration of use (range, SD) (months)	87(2–238, SD 119)

Values are means unless other specified, with range and standard deviation between parantheses

Suppression of HPA axis was present in three out of twelve individuals. The cortisol values are shown in Table 2. The corticosteroids they used were nasal beclomethasone, inhalation beclomethasone and nasal plus inhalation fluticasone, respectively. None of the affected individuals used ritonavir or cobicistat. There were also no other CYP3A4 inhibitors or inducers used by any of the patients.

**Table 2:** Cortisol values during ACTH stimulation test

Patient	Kind of corticosteroid	Duration of use of corticosteroid (months)	Morning cortisol (nmol/L)	Cortisol 60 min after ACTH injection (nmol/L)	Cortisol 90 min after ACTH injection (nmol/L)
1	Fluticasone (I)	48	320	750	770
2	Beclomethasone (I) and Momethasone (N)	36	100	580	650
3	Momethasone (N)	2	390	650	700
4	Beclomethasone (I)	72	340	740	880
5	Beclomethasone (N)	**240**	**410**	**460**	**510**
6	Beclomethasone (I)	**1**	**200**	**400**	**440**
7	Budesonide (I)	72	270	610	640
8	Fluticasone (I and N)	**60**	**140**	**380**	**410**
	Persons with booster				
9	Beclomethasone (N)	3	420	770	820
10	Beclomethasone (I)	36	250	670	790
11	Ciclesonide (I)	24	307	634	705
12	Beclomethasone (I) and fluticasone (N)	240	300	567	595

The bold values are of the patients with HPA-axis suppression, (N between parentheses means nasal corticosteroid and I means inhalation corticosteroid). Patient 6 had switched to this corticosteroid a month previously, but had used another corticosteroid before that. Patients 11 and 12 received intramuscular injections of ACTH, whereas the other patients received intravenous injections

## Discussion

In this study we identified only 12 patients treated for HIV who also used inhalation or nasal cortocosteroids. Because of this low number of patients it is impossible to draw definitive conclusions. In our study HPA axis suppression was found in three of twelve HIV-infected persons who used different dosages of inhalation or nasal corticosteroids: two of them used beclomethasone and the other fluticasone. All three patients were asymptomatic for signs of HPA axis suppression and none of them used a CYP3A4 inhibitor.

This is the first study in which HIV-patients on inhaled or nasal corticosteroids were screened for HPA axis suppression. These patients were selected by their medication use, not because of symptoms. Several case reports and case series have been published earlier in which symptomatic patients on inhalation or nasal corticosteroids had HPA axis suppression [3].

In a meta-analysis HPA axis suppression was seen in 4.2% (95% CI 0.5–28.9%) in general patients using nasal corticosteroids and in 7.8% of patients taking inhaled corticosteroids (95% CI 4.2–13) [1]. We found suppression in 3 out of 12 individuals in this study. Although on first sight this proportion is high, we realize that the group we studied is very small and we used a relatively strict definition for HPA axis suppression. (Morning cortisol below 80 nmol/L and cortisol below 550 nmol/L during stimulation with 250 microg ACTH). In the meta-analysis the HPA axis was assessed by insulin tolerance test, metyrapone test, CRH test or an ACTH stimulation test. HPA axis suppression was defined as a stimulated cortisol of 500 nmol/L or less. One of our patients had a value of 460 nmol/L at 30 min and 510 nmol/L after 90 min so would have a normal HPA axis by this alternative definition. However a recent study proved that one should use assay specific cutoff values. We used the Roche assay for which a cut off value of 550 nmol/L is reasonable [6].

One study of healthy volunteers showed that beclomethasone in combination with ritonavir does not lead to adrenal suppression tested by an ACTH-stimulation test [7]. This study and pharmacokinetic evidence has resulted in the general advice to change the inhalation corticosteroid fluticasone to beclomethasone if a patient also uses a CYP3A4 inhibitor [3, 4] This advice was already actively implemented in the involved hospitals. Therefore we were unable to assess whether HPA axis suppression is seen more often in patients using a CYP3A4 inhibitor together with a local glucocorticoid metabolized by CYP3A4.

In 2016, Elliot et al. gave recommendations for physicians concerning CYP3A4 inhibitors and use of corticosteroids [8]. They advise to switch inhaled steroids to beclomethasone or a steroid sparing agent and then determine a 9 am cortisol. If this cortisol is lower than 450 nmol/L the next step will be an ACTH stimulation test. If the cortisol is below 550 nmol/L during this ACTH stimulation test, their advice is to start steroid replacement. Their emphasis on increased awareness for iatrogenic HPA axis suppression is important, as is the collaboration between HIV specialists and endocrinologists. However their recommendations are not based on large studies and HPA axis suppression is also seen in patients not using CYP3A4 inhibitors.

## Conclusion

In our small scale study HPA axis suppression was found in 3 out of 12 screened patients, none of them used a CYP3A4 inhibitor. This demonstrates that HPA axis suppression can occur in every patient on nasal or inhaled corticosteroids.
